# Left Heart Chamber Volumetric Assessment by Automated Three-Dimensional Echocardiography in Heart Transplant Recipients

**DOI:** 10.3389/fcvm.2022.877051

**Published:** 2022-04-27

**Authors:** Yiwei Zhang, Chun Wu, Wei Sun, Shuangshuang Zhu, Yanting Zhang, Yuji Xie, Ye Zhu, Zisang Zhang, Yang Zhao, Yuman Li, Mingxing Xie, Li Zhang

**Affiliations:** ^1^Department of Ultrasound Union Hospital, Tongji Medical College, Huazhong University of Science and Technology, Wuhan, China; ^2^Hubei Province Key Laboratory of Molecular Imaging, Wuhan, China

**Keywords:** 3D echocardiography, heart transplant, heart model, left atrial volume, left ventricular function, left ventricular volume

## Abstract

**Background:**

Recently, a new automated software (Heart Model) was developed to obtain three-dimensional (3D) left heart chamber volumes. The aim of this study was to verify the feasibility and accuracy of the automated 3D echocardiographic algorithm in heart transplant (HTx) patients. Conventional manual 3D transthoracic echocardiographic (TTE) tracings and cardiac magnetic resonance (CMR) images were used as a reference for comparison.

**Methods:**

This study enrolled 103 healthy HTx patients prospectively. In protocol 1, left ventricular end-diastolic volume (LVEDV), LV end-systolic volume (LVESV), left atrial max volume (LAVmax), LA minimum volume (LAVmin) and LV ejection fraction (LVEF) were obtained using the automated 3D echocardiography (3DE) and compared with corresponding values obtained through the manual 3DE. In protocol 2, 28 patients’ automated 3DE measurements were compared with CMR reference values. The impacts of contour edit and surgical technique were also tested.

**Results:**

Heart Model was feasible in 97.1% of the data sets. In protocol 1, there was strong correlation between 3DE and manual 3DE for all the parameters (*r* = 0.77 to 0.96, p<0.01). Compared to values obtained through manual measurements, LV volumes and LVEF were overestimated by the automated algorithm and LA volumes were underestimated. All the biases were small except for that of LAVmin. After contour adjustment, the biases reduced and all the limits of agreement were clinically acceptable. In protocol 2, the correlations for LV and LA volumes were strong between automated 3DE with contour edit and CMR (*r* = 0.74 to 0.93, p<0.01) but correlation for LVEF remained moderate (*r* = 0.65, *p* < 0.01). Automated 3DE overestimated LV volumes but underestimated LVEF and LA volumes compared with CMR. The limits of agreement were clinically acceptable only for LVEDV and LAVmax.

**Conclusion:**

Simultaneous quantification of left heart volumes and LVEF with the automated Heart Model program is rapid, feasible and to a great degree it is accurate in HTx recipients. Nevertheless, only LVEDV and LAVmax measured by automated 3DE with contour edit seem applicable for clinical practice when compared with CMR. Automated 3DE for HTx recipients is a worthy attempt, though further verification and optimization are needed.

## Introduction

Orthotopic heart transplantation (HTx) is one of the most effective treatments for patients with end-stage heart disease. With improvement in operative techniques and postoperative surveillance and therapy, the median survival after adult heart transplants has increased to 12.5 years (1–4). Previous studies have shown that the volume of left ventricular (LV) and left atrial(LA) are crucially related to overall left heart function (5–7), which is of great importance for the assessment of transplanted heart. Echocardiography has become post-transplantation annual routine follow-up for its convenience and accuracy and usually used for the assessment of heart volumes. 3-dimensional (3D) transthoracic echocardiographic (TTE) measurements of cardiac chamber volumes are proved superior to 2-dimensional (2D) techniques in accuracy and reproducibility, due to avoidance of geometric assumptions and foreshortened views (5–7). However, widespread use of 3D TTE for LA and LV volume assessments has not become a clinical reality, as time and training are required to obtain accurate and reproducible 3DE volumetric measurements (5, 8, 9).

Heart Model is a novel automated 3DE software with the ability of simultaneous quantification of heart chamber volumes and LV ejection fraction (LVEF) within few seconds. Previous studies have shown the feasibility and accuracy of Heart Model in measuring left heart volumes and LVEF in multiple cohorts (10–12). Nevertheless, this automated adaptive analytics algorithm relies on the 3DE database comprised of morphologies derived from a ‘training’ population, which may not adequately encompass the HTx recipients cohort, whose heart geometry is usually grossly distorted (13).

Thus, the aim of this study was to explore the accuracy and reproducibility of the Heart Model program for automated measurement of LV, LA volumes and LVEF from 3DE datasets in the HTx recipients, using expert manual 3DE and cardiac magnetic resonance (CMR) as references.

## Materials and Methods

### Study Population

A total of 103 HTx patients at Union Hospital in Wuhan, China, were prospectively enrolled in this study between January 2018 and January 2020.

In Protocol 1, we prospectively included 103 HTx patients referred to the echocardiography laboratory for their routine follow-up examination. All of them presented as clinically well and underwent 2D and 3D TTE. 3 of the 103 patients were excluded for poor 3D-echocardiographic image quality unsuitable for automated analysis. LV end-diastolic volume (LVEDV), LV end-systolic volume (LVESV), LVEF, LA max volume (LAV max) and LA minimum volume (LAV min) derived from automated 3DE were compared with the manual 3DE and 2D biplane Simpson method measurements.

In Protocol 2, 28 of the 103 HTx recipients who agreed to undergo CMR examination within the following 24 h after echocardiographic examination were enrolled. The automated 3D echocardiographic measurements of LVEDV, LVESV, LVEF, and LAV were compared with the CMR values. 28 participants were divided into biatrial group and bicaval group according to the surgical technique and the correlation coefficients between automated 3DE measurements and CMR measurements of the two groups were compared.

Weight, height, heart rate, primary diagnosis, surgical technique and time since HTx of every patients were recorded. The study was approved by the Ethics Committee of Tongji Medical College, Huazhong University of Science and Technology. Written informed consent of all participants have been obtained.

### Echocardiographic Image Acquisition

All echocardiographic examinations were performed by an experienced echocardiographic doctor using EPIQ 7C (Philips Medical Systems) and an X5-1 matrix probe (Philips Medical Systems) with the patient breath-holding. 2D echocardiographic (2DE) images were acquired from the parasternal short-axis view at the apical four-, three-, and two- chamber views. Foreshortening of the left ventricle and left atrium has been avoided. 3D echocardiographic acquisitions were recorded from the four-chamber apical view in heart model mode, and were gathered over four cardiac cycles, during a breath-hold lasting for a few seconds (14). The volume rate was adjusted above 18 Hz when 3D echocardiographic acquisition was performed. Imaging settings were optimized for visualizing endocardium before every acquisition.

### Two-Dimensional Echocardiography Analysis

LV end-diastolic volume (LVEDV), LV end-systolic volume (LVESV), LVEF, LA max volume (LAV max) and LA minimum volume (LAV min) were calculated using the biplane Simpson method, by means of a commercially available software (QLAB-2DQ, Philips Healthcare). The papillary muscles were included in the LV cavity when tracking the endocardial contours.

### Manual (Semiautomated) Three-Dimensional Echocardiography Analysis

For manual 3D echocardiographic analysis, a semi-automatically derived 3D echocardiographic method was used. Operators used commercially available software (QLAB-3DQadv, Philips Healthcare) to measure LVEDV, LVESV, LVEF, LAVmax and LAVmin. Firstly, the multiplanar views were adjusted to optimize the horizontal and vertical lines in the middle of LV chamber. Then the operator placed reference points at the end-diastolic and end-systolic frames: two points to identify the mitral valve annulus and the apex in four- and two- chamber view. For LA, this included two points to identify the mitral valve annulus in each of the two apical views, and one point to identify the center of the posterior wall in either view. Finally, the software automatically identified LV and LA endocardial border and created a 3D model of left cardiac chamber to calculate LV, LA volumes and LVEF.

### Automated Three-Dimensional Echocardiography Analysis

**FIGURE 1:**
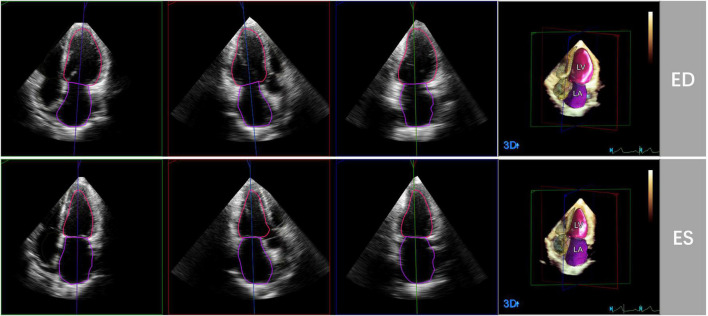
Representative case of automated 3D echocardiographic analysis for left heart chamber quantification. Left heart chambers’ endocardial borders were automatically detected by the Heart Model software at end-diastole (ED) and end-systole (ES) in apical four-, three-, and two chamber sections.

3D echocardiographic acquisitions were also analyzed by the HeartModel software. This algorithm is able to automatically detect LV and LA endocardial borders at end-diastole and end-systole and measure LVEDV, LVESV, LVEF, LAVmax and LAVmin (Figure 1). Observers can freely move the adjustable slider to optimize cardiac chamber border identification according to their preference, including global and regional editing.

### Cardiac Magnetic Resonance Imaging

Cardiac magnetic resonance was performed in 28 of 103 patients within 24 hours of the echocardiography by 1.5-Tesla system (MAGNETOM Aera, Siemens Healthineers, Erlangen, Germany). In each patient, the long axis of the heart was identified by retrospective electrocardiogram-gated localizing spin-echo sequences. Steady state free-precession dynamic gradient echo cine loops of the left ventricle and left atrium were then acquired during 10- to 15-second breath-holds. The cine image parameters in our study were obtained as follows: slice thickness of 8 mm, matrix of 205 × 256 pixels, and flip angle of 80°.

### Cardiac Magnetic Resonance Analysis

Cardiac magnetic resonance images were analyzed using commercial software (Argus, Siemens Healthineers). Left cardiac volumetric and functional parameters were derived by manual delineation of the endocardial contours on the continuous LV and LA short-axis cine images at the end-diastolic frame and end-systolic frame. Papillary muscles and trabeculations were included in the LV cavity, while pulmonary veins and LA appendage were excluded from the LA cavity.

### Reproducibility

Of the 100 participants in protocol 1, 20 participants were selected randomly for the evaluation of the reproducibility of manual 3DE and automated 3DE. For test-retest variability, the same observer analyzed 3D echocardiographic data sets of each patient. For intraobserver variability, the same observer analyzed the 3D echocardiographic data set 2 weeks later after the first analysis, blinded to the previous measurements. For interobserver variability, two blinded and independent observers analyzed the 3D echocardiographic data set.

### Statistical Analysis

Continuous variables were presented as mean ± SD. and nominal variables as percentages. 2D and 3D echocardiographic images were analyzed offline by a single investigator who was blind to the values of echocardiographic and CMR measurements. CMR measurements were performed by an observer experienced in CMR analysis, who was not allowed to view the echocardiographic results. Pearson’s or Spearman’s correlation coefficient and Bland-Altman analysis were used to test the correlation and agreement between two sets of measurements by calculating the bias (mean difference) and the limits of agreement (LOA; 1.96 SDs around the mean difference). The descriptions of the strength of correlations were based on the following standard: r value between 0.7 and 0.9 was considered strong correlation; r value between 0.5 and 0.69 was considered moderate correlation; and r value between 0.3 and 0.5 was considered weak correlation. For LV and LA volumes, the relative bias and the percentage error of the LOA were also calculated. The reference method in protocol 1 and protocol 2 was manual 3DE and CMR, respectively. The LOA is used to estimate the precision or random error of the measurements around the bias. A percentage error of the LOA below 30% was considered clinically acceptable.

For 3D echocardiographic measurements, intraobserver, and interobserver variability was examined and expressed as coefficient of variation (the absolute difference between two measurements in percentage of their mean in each patient and then averaged over the entire study group). Comparisons of correlation coefficients were performed on MedCalc version 18.2.1 (MedCalc Software, Ostend, Belgium). All statistical analyses were performed on SPSS version 22.0 (Statistical Package for the Social Sciences, Chicago, Illinois), GraphPad Prism version 8.0.1 and MedCalc version 18.2.1 (MedCalc Software, Ostend, Belgium). A *P*-value <0.05 was considered statistically significant.

## Results

### Study Population

103 HTx recipients (80 male, 23 female) were included in protocol 1. 3 of them were excluded from analysis because of failure to be analyzed by automated 3D echocardiographic system, with the remaining 100 participants included in the final analysis. The feasibility of automated 3D echocardiographic system in protocol 1 was 97.1%, and feasibility of 2D echocardiographic analysis and manual 3D echocardiographic analysis were both 100.0%. 28 HTx recipients (20 male, 8 female) were enrolled in protocol 2. The feasibility of both automated 3D echocardiographic system and CMR in protocol 2 were 100.0%. The baseline clinical characteristics of all participants, including those in protocol 1 and protocol 2, are summarized in Table 1. The mean values of LVEDV, LVESV, LVEF, LAVmax and LAVmin measured by different methods are presented in Table 2.

**TABLE 1:** Clinical characteristics of the study subjects.

Variable	Protocol 1	Protocol 2
Number of patients	100	28
Gender, male	79 (79)	20 (71)
Age, years	47.3 ± 12.7	45.6 ± 13.5
BSA, m^2^	1.69 ± 0.17	1.64 ± 0.17
Heart rate, bpm	87 ± 9	88 ± 7
**Primary diagnosis**		
DCM	56 (56)	13 (46)
CAD	15 (15)	5 (18)
VHD	8 (8)	1 (4)
Others	21 (21)	9 (32)
**Surgical technique**		
Biatrial	39 (39)	12 (43)
Bicaval	61 (61)	16 (57)
Time since transplantation, months	22.2 ± 24.1	19.3 ± 28.1
%HM feasibility	97.1	100.0

*DCM, dilated cardiomyopathy; CAD, coronary artery disease; VHD, valvular heart disease; HM, automated 3DE by Heart Model.*

**TABLE 2:** Mean of LV volumes, LVEF and LA volumes obtained by the different methods.

Method	n	LVEDV (mL)	LVESV (mL)	LVEF (%)	LAVmax (mL)	LAVmin (mL)
**Protocol 1**						
2DE	103	86.9 ± 20.8	34.3 ± 9.4	60.5 ± 5.1	88.0 ± 24.3	53.3 ± 19.6
**Automated 3DE**						
Without contour edit	100	100.1 ± 23.3	37.4 ± 11.6	62.9 ± 5.8	84.0 ± 28.6	51.7 ± 23.1
With contour edit	100	94.9 ± 21.9	37.0 ± 10.4	61.0 ± 5.1	85.6 ± 25.0	56.5 ± 20.8
Manual 3DE	103	90.0 ± 21.2	36.1 ± 10.2	60.0 ± 5.0	83.9 ± 23.6	57.0 ± 20.0
**Protocol 2**						
**Automated 3DE**						
Without contour edit	28	97.8 ± 23.6	39.0 ± 13.1	60.8 ± 5.6	75.3 ± 23.8	46.1 ± 15.3
With contour edit	28	88.4 ± 20.8	35.5 ± 11.8	60.0 ± 6.2	84.5 ± 22.6	57.2 ± 17.4
CMR	28	85.2 ± 21.0	33.9 ± 12.0	60.8 ± 6.3	89.3 ± 23.6	76.7 ± 22.4

### Automated Three-Dimensional Echocardiography Versus Manual Three-Dimensional Echocardiography

There were strong correlations for LVEDV, LVESV, LAVmax and LAVmin between automated 3DE and manual 3DE (*r* = 0.87, *r* = 0.84, *r* = 0.90 and *r* = 0.83, respectively, *P* < 0.01 for all). The automated 3DE measurements of LVEF correlated moderately with the reference value measured by manual 3DE (*r* = 0.79, *P* < 0.01). After contour edit, the correlations for volumes and LVEF were all excellent (*r* = 0.95 for LVEDV, *r* = 0.92 for LVESV, *r* = 0.77 for LVEF, *r* = 0.96 for LAVmax, and *r* = 0.94 for LAVmin, *P* < 0.01 for all). Results are presented in Table 3.

**TABLE 3:** Comparison of LV volumes, LVEF, and LA volumes measured by 2DE, automated 3DE against manual 3D echocardiographic measurements.

Method	Parameter	r	*P*	Bias ± LOA	Relative bias (%)	Percentage error (%)
2DE						
	LVEDV (mL)	0.88	<0.01	−3.1 ± 17.8	–3.0	20.1
	LVESV (mL)	0.82	<0.01	−1.9 ± 10.7	3.9	30.4
	LVEF (%)	0.67	<0.01	0.5 ± 7.9	−	–
	LAVmax (mL)	0.91	<0.01	4.0 ± 19.8	5.5	23.0
	LAVmin (mL)	0.88	<0.01	−3.8 ± 18.9	5.1	34.3
**Automated 3DE**						
Without contour edit	LVEDV (mL)	0.87	<0.01	10.1 ± 21.9	12.1	23.0
	LVESV (mL)	0.84	<0.01	1.3 ± 11.1	3.9	30.2
	LVEF (%)	0.79	<0.01	2.9 ± 6.5	−	–
	LAVmax (mL)	0.90	<0.01	0.0 ± 24.0	0.7	28.6
	LAVmin (mL)	0.83	<0.01	−5.3 ± 24.6	–9.8	45.3
With contour edit	LVEDV (mL)	0.95*	<0.01	4.8 ± 11.7	5.7	12.7
	LVESV (mL)	0.92*	<0.01	0.9 ± 7.2	3.1	19.7
	LVEF (%)	0.77	<0.01	1.0 ± 5.6	−	–
	LAVmax (mL)	0.96*	<0.01	1.7 ± 14.2	1.9	16.7
	LAVmin (mL)	0.94*	<0.01	−0.6 ± 14.1	–0.7	24.8

Relative bias = (parameter _method_-parameter_manual 3DE_)/parameter _manual 3DE_. Percentage error = LOA/mean value of parameter measured by studied method and manual 3DE. *, p < 0.05 compared with 2DE.

**FIGURE 2:**
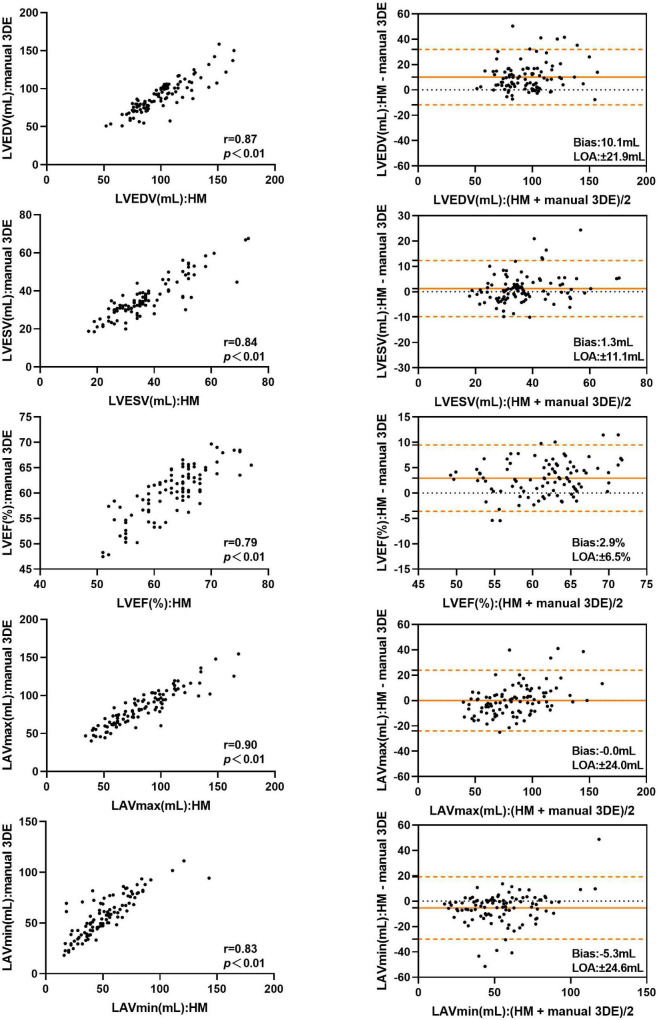
Comparison between automated 3DE without contour adjustment and manual 3DE of left heart volumes and ejection fraction: correlation and Bland-Altman analysis. HM: Automated 3DE by Heart Model without contour adjustment.

Compared with the manual 3DE reference values, LVEDV, LVESV, LVEF, and LAVmax, without contour edit, were overestimated by the automated 3DE, with tolerable biases (10.1 mL for LVEDV, 1.3 ml for LVESV, 2.9% for LVEF, and 0.0 ml for LAVmax) between the two methods (Figure 2). Automated 3DE without contour adjustment underestimated LAVmin compared with manual 3DE, with small bias (−5.3 ml). When there was no contour adjustment, the automated 3DE measurements of LVEDV were on average 12.1% higher than values derived by manual 3DE, while automated 3DE-derived LAVmax was on average 0.7% lower than values obtained by manual 3DE (relative biases). The LOA for both were clinically acceptable (percentage error of the LOA<30%), while that of LVESV and LAVmin were not (Table 3).

**FIGURE 3:**
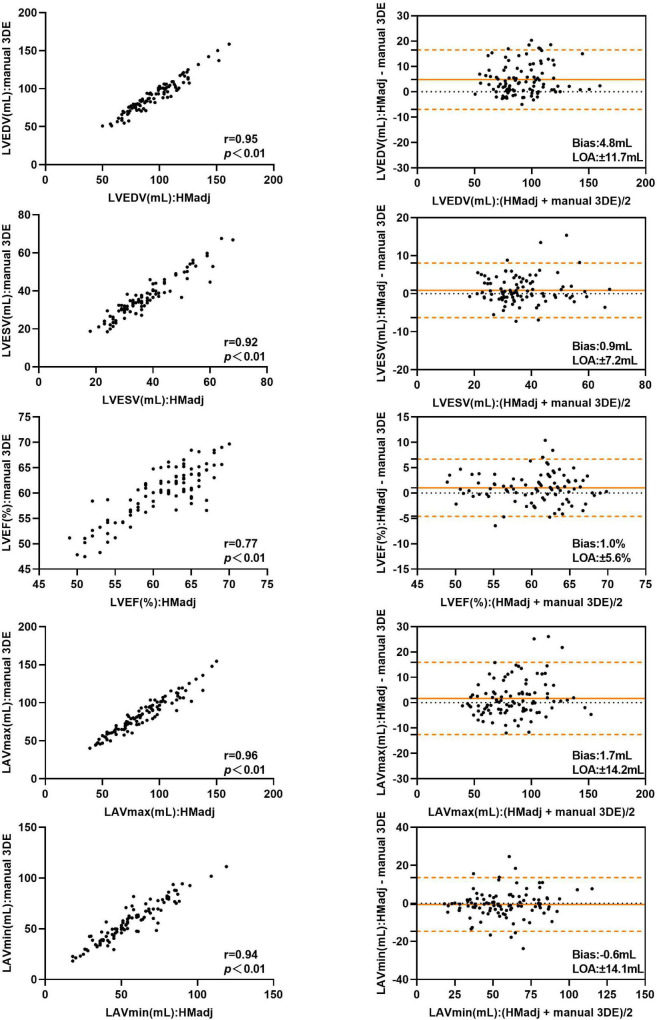
Comparison between automated 3DE with contour adjustment and manual 3DE of left heart volumes and ejection fraction: correlation and Bland-Altman analysis. HMadj: Automated 3DE by Heart Model with contour adjustment.

After contour edit, the biases and LOA for LVEDV, LVESV, LVEF and LAVmin between automated 3DE and manual 3DE were reduced (Figure 3). The automated 3DE measurements of LV volumes and LVEF were overestimated compared with those of manual 3DE, with small biases (biases, 4.8 ml for LVEDV, 0.9 ml for LVESV, and 1.0% for LVEF; relative bias, 5.7% for LVEDV, 3.1% for LVESV of manual 3DE values). LAVmin obtained by automated 3DE was underestimated, with negligible bias (bias, −0.6 ml, relative bias, −0.7% of manual 3D echocardiographic values). However, bias for LAVmax compared with manual 3DE increased when contour edit was performed. LAVmax obtained by automated 3DE with contour adjustment was slightly overestimated with small bias (1.7 ml). All the LOA were clinically acceptable (Table 3).

### Two-Dimensional Echocardiography Versus Manual Three-Dimensional Echocardiography

Detailed results are presented in Table 3. The correlations for LV, LA volumes between 2DE and manual 3DE were both strong with no significant difference between automated 3DE measurements and manual 3DE ones (*P* > 0.05). However, when contour adjustments were performed, the correlations for left cardiac chamber volumes between automated 3DE and manual 3DE were significantly stronger than those between 2DE and manual 3DE. 2DE measured LVEF correlated moderately with values derived from manual 3DE, while the correlations for LVEF between automated 3DE and manual 3DE, with or without contour adjustment, were strong. There was no significant difference between those correlations.

Compared with manual 3DE, 2DE underestimated LVEDV, LVESV, and LAVmin but overestimated LVEF and LAVmax. In general, the biases in measurements of LVESV, LAVmax were smaller for automated 3DE than 2DE. For automated 3DE with contour edit, the biases in measurements of all parameters were smaller than 2DE, except for LVEDV and LVEF. The LOA of automated 3DE with contour edit were tighter than those derived from 2DE.

### Automated Three-Dimensional Echocardiography Versus Cardiac Magnetic Resonance

Table 4 represents the details of the comparisons between the automated 3DE echocardiographic measurements and the corresponding values obtained by CMR. There was strong correlation for LAVmax and modest correlations for LVEDV, LVESV, LVEF, and LAVmin between automated 3DE without contour edit and CMR (*r* = 0.77, *r* = 0.68, *r* = 0.63, *r* = 0.62, and *r* = 0.64, respectively, *P* < 0.01 for all). The correlations for LV and LA volumes were strong between automated 3DE with contour edit and CMR (*r* = 0.92 for LVEDV, *r* = 0.74 for LVESV, *r* = 0.93 for LAVmax, *r* = 0.79 for LAVmin, *P* < 0.01 for all), while correlation for LVEF remained moderate (*r* = 0.65, *P <* 0.01).

**TABLE 4:** Comparison of LV volumes, LVEF, and LAV measured by automated 3DE against CMR measurments.

Method	Parameter	r	*P*	Bias ± LOA	Relative bias (%)	Percentage error (%)
**Automated 3DE**						
Without contour edit	LVEDV (mL)	0.68	<0.01	12.6 ± 30.4	17.0	33.2
	LVESV (mL)	0.63	<0.01	5.1 ± 18.5	19.9	54.7
	LVEF (%)	0.62	<0.01	0.0 ± 10.2	−	–
	LAVmax (mL)	0.77	<0.01	−14.1 ± 31.6	–15.4	38.4
	LAVmin (mL)	0.64	<0.01	−30.6 ± 33.9	–38.9	55.1
With contour edit	LVEDV (mL)	0.92*	<0.01	3.2 ± 13.7	4.5	15.8
	LVESV (mL)	0.74	<0.01	1.6 ± 12.7	8.0	36.7
	LVEF (%)	0.65	<0.01	−0.8 ± 10.2	−	–
	LAVmax (mL)	0.93*	<0.01	−4.8 ± 17.0	–5.0	19.5
	LAVmin (mL)	0.79	<0.01	−19.5 ± 27.1	–24.7	40.5

Relative bias = (parameter _method_-parameter _CMR_)/parameter _CMR_. Percentage error = LOA/mean value of parameter measured by studied method and CMR. *p < 0.05 compared with without contour edit group.

**FIGURE 4:**
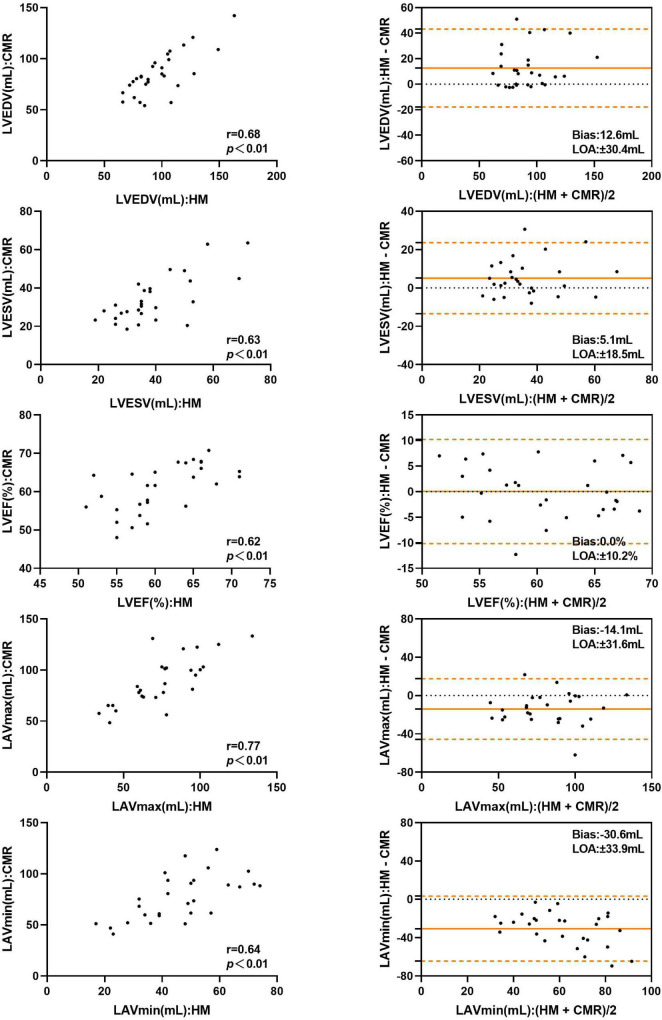
Comparison between automated 3DE without contour adjustment and CMR of left heart volumes and ejection fraction: correlation and Bland-Altman analysis. HM: Automated 3DE by Heart Model without contour adjustment.

The LVEDV and LVESV derived by automated 3DE without contour edit were overestimated compared with CMR reference values, with small bias. The LAVmax and LAVmin obtained by automated 3DE without contour edit were underestimated with big bias (Figure 4). The LOA were wide for all (Table 4).

**FIGURE 5:**
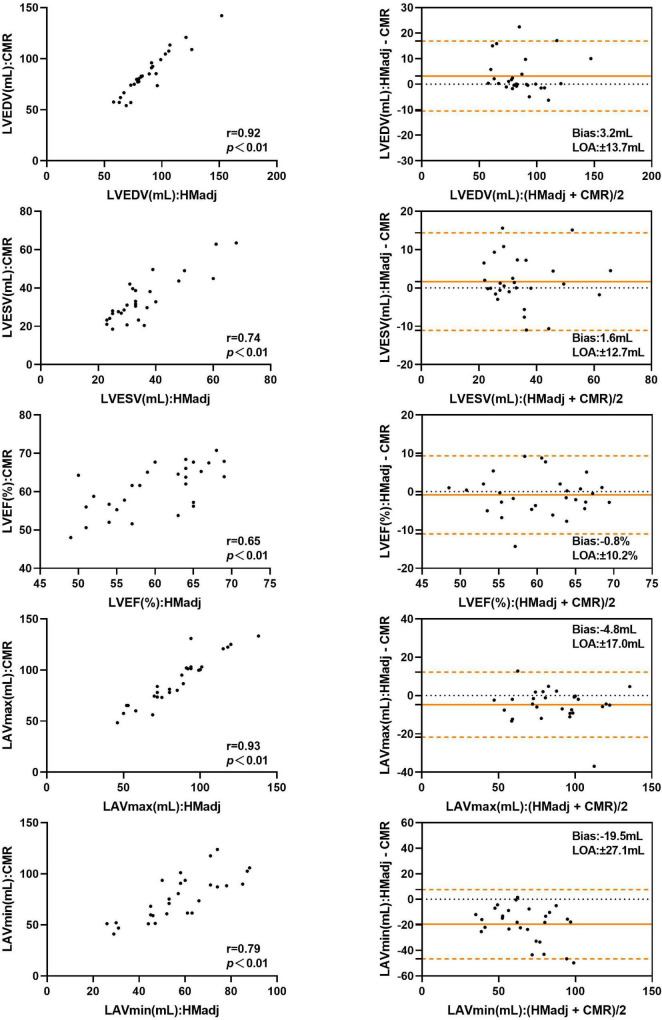
Comparison between automated 3DE with contour adjustment and CMR of left heart volumes and ejection fraction: correlation and Bland-Altman analysis. HMadj: Automated 3DE by Heart Model with contour adjustment.

With contour adjustment of automated 3DE values, the bias for LV and LA volumes compared with CMR were reduced (Figure 5). Automated 3DE with contour edit slightly overestimated LVEDV and LVESV (biases, 3.2 ml for LVEDV, 1.6 ml for LVESV; relative bias, 4.5% for LVEDV, 8.0% for LVESV of CMR values). The LAVmax and LAVmin were underestimated by automated 3DE with contour edit (biases, −4.8 ml for LAVmax, −19.5 ml for LAVmin; relative bias, −5.0% for LAVmax, −24.7% for LAVmin of CMR values). The LOA for LV and LA volumes were also reduced but were clinically acceptable only for LVEDV and LAVmax (Table 4).

When the impact of surgical technique was assessed, the biatrial group’s correlations of LVESV and LVEF derived from automated 3DE with CMR reference values were stronger than those of bicaval group with contour edit, and the difference was statistically significant (Table 5).

**TABLE 5:** Effect of surgical technique on measurements from the automated 3DE compared with CMR measurements.

	n	Automated 3DE	CMR	r	Bias ± LOA
**LVEDV (mL)**					
**Without contour edit**					
Biatrial	12	94.5 ± 19.1	85.9 ± 15.5	0.75	−8.6 ± 24.9
Bicaval	16	100.3 ± 26.9	84.7 ± 24.8	0.78	−15.6 ± 33.7
**With contour edit**					
Biatrial	12	86.6 ± 15.4	85.9 ± 15.5	0.98	−0.7 ± 6.0
Bicaval	16	89.8 ± 24.5	84.7 ± 24.8	0.94	−5.1 ± 16.6
**LVESV (mL)**					
**Without contour edit**					
Biatrial	12	37.3 ± 11.2	33.7 ± 11.2	0.82	−3.6 ± 13.6
Bicaval	16	40.3 ± 14.6	34.0 ± 12.9	0.68	−6.2 ± 21.7
**With contour edit**					
Biatrial	12	34.3 ± 11.1	33.7 ± 11.2	0.95*	−0.6 ± 6.4
Bicaval	16	36.4 ± 12.6	34.0 ± 12.9	0.66	−2.4 ± 16.0
**LVEF (%)**					
**Without contour edit**					
Biatrial	12	61.3 ± 5.6	61.2 ± 7.8	0.74	−0.1 ± 10.3
Bicaval	16	60.5 ± 5.7	60.5 ± 5.1	0.52	−0.0 ± 10.4
**With contour edit**					
Biatrial	12	60.5 ± 7.1	61.2 ± 7.8	0.93*	0.7 ± 5.6
Bicaval	16	59.6 ± 5.7	60.5 ± 5.1	0.28	0.9 ± 12.8
**LAVmax (mL)**					
**Without contour edit**					
Biatrial	12	89.1 ± 22.5	99.7 ± 19.6	0.82	10.6 ± 25.1
Bicaval	16	64.9 ± 19.4	81.5 ± 23.9	0.67	16.6 ± 35.6
**With contour edit**					
Biatrial	12	96.5 ± 20.2	99.7 ± 19.6	0.97	3.2 ± 9.6
Bicaval	16	75.6 ± 20.5	81.5 ± 23.9	0.90	5.9 ± 20.9
**LAVmin (mL)**					
**Without contour edit**					
Biatrial	12	49.1 ± 12.9	85.8 ± 19.2	0.60	36.7 ± 25.6
Bicaval	16	43.9 ± 16.9	69.9 ± 22.7	0.64	26.0 ± 34.6
**With contour edit**					
Biatrial	12	60.0 ± 13.7	85.8 ± 19.2	0.72	25.8 ± 25.9
Bicaval	16	55.1 ± 19.9	69.9 ± 22.7	0.83	14.78 ± 24.9

*p < 0.05 compared with bicaval group.

### Reproducibility of Three-Dimensional Echocardiographic Measurements

Intraobserver and interobserver (with or without contour edit) variability for 3D echocardiographic measurements of LV, LA volumes and LVEF is summarized in Table 6.

**TABLE 6:** Test-retest, intraobserver, interobserver variability (coefficients of variation) for the automated and manual 3D echocardiographic masurements of LV volumes, LVEF and LA volumes.

	Automated 3DE				Manual 3DE	
	Test-retest without contour edit (%)	Test-retest with contour edit (%)	Interobserver without contour edit (%)	Interobserver with contour edit (%)	Intraobserver (%)	Interobserver (%)
LVEDV	4.0 ± 5.3	9.4 ± 7.9	4.9 ± 5.6	8.0 ± 8.9	10.9 ± 11.3	10.3 ± 13.6
LVESV	8.5 ± 11.3	12.7 ± 8.8	6.2 ± 9.0	11.7 ± 10.4	13.9 ± 14.5	17.1 ± 13.9
LVEF	3.6 ± 5.7	5.6 ± 4.6	4.1 ± 3.9	5.6 ± 4.5	8.4 ± 7.0	7.9 ± 5.6
LAVmax	8.3 ± 10.1	11.5 ± 9.4	8.3 ± 9.9	9.8 ± 10.0	13.1 ± 12.0	14.8 ± 13.7
LAVmin	7.1 ± 9.0	13.5 ± 12.5	6.4 ± 8.8	15.8 ± 10.2	13.4 ± 11.6	15.6 ± 9.8

Intraobserver and interobserver reproducibility of automated 3DE was high without contour edit (variability value <10%). When contour adjustment was performed, the variability values increased, however, those of LVEDV and LVEF remained low. As for manual 3DE, variability values of intraobserver and interobserver were higher than that of automated 3DE, no matter whether the contour adjustment was performed or not.

## Discussion

To the best of our knowledge, this is the first study to assess the automated 3D echocardiographic algorithm (Heart Model) for quantification of left cardiac chamber volumes and LVEF in HTx recipients. Previous studies have demonstrated feasibility and reproducibility of automated 3DE in measuring left heart chamber and its function. In these studies, automated 3DE measurements have shown strong correlations with values derived from 2DE, manual 3DE, and CMR (11, 15, 16). These results were widely verified in adults, children, healthy population, and patients with specific diseases, including mitral regurgitation or atrial fibrillation (10, 17, 18). However, a transplanted heart is different from a normal one in cardiac anatomy, including its special location and structure after the orthotopic transplantation. Therefore, we tested the feasibility and accuracy of the automated 3DE technique, HeartModel, in HTx recipients.

Our major findings are as follows: in HTx recipients, (1) the correlations for LV and LA volumes between automated 3DE and manual 3DE were strong, while the correlation of LVEF between the two was moderate. After contour adjustment, all the values derived from automated 3DE showed strong correlations with manual 3DE reference values; (2) All the automated 3DE measurements had stronger correlations with manual 3DE reference values than 2DE-derived values except for LVEF; (3) LV volumes, LAVmin, and LVEF derived from automated 3DE were moderately correlated with CMR reference values, but the automated 3DE measurements of LAVmax correlated strongly with CMR reference values. When contour edit was performed, all the correlations became strong except for LVEF; and (4) surgical techniques had no impact on the correlations for most left cardiac chamber volumes between automated 3DE and CMR whether or not contour edit was performed. Only the biatrial group’s correlations for LVESV and LVEF between automated 3DE and CMR were stronger than those of bicaval group after contour edit.

In our study, automated 3DE presented a high degree of feasibility in HTx recipients, which was in line with previous studies performed in unselected patients. Although the position of the transplanted heart is more or less different from that of the normal heart, HeartModel can overcome this barrier in its automatic analysis.

However, the correlations between automated 3DE measurements and manual 3DE measurements for left heart chamber and LVEF in our cohort were lower than those obtained in previous studies. Contour adjustment can improve the correlations but it is hard to reach the level found in patients without HTx. Nevertheless, the correlations for left cardiac chamber volumes between automated 3DE and manual 3DE were stronger than those between 2DE and manual 3DE. 3DE is independent of geometric assumptions, which makes it more reliable than 2DE, and this can explain the stronger correlations between automated 3DE measurements and manual 3DE ones.

When automated 3DE was compared with the gold standard, CMR, only the accuracy of automated 3DE measurements of LVEDV and LAVmax was similar to that of previous studies after the contour edit (11). We believe this poor precision could be explained by the workflow of Heart Model and the distorted anatomy of transplanted hearts. The first step of Heart Model’s automated analysis is knowledge-based identification, which is trained to use approximately 1000 echo images from a wide variety of heart shapes and sizes. The software screens the cardiac chamber shapes, including the overall morphological size, shape, curvature, and volume of the 3DE data, to select the best “matching” shapes (11). However, a transplanted heart was made up of the donor’s and part of the recipient heart. It is hard to find an appropriate “matching” shape in the database. The moderate precision of automated 3DE for LVESV measurements can explain the moderate correlation for LVEF between automated 3DE and CMR. This was also reported in some previous publications (15, 17). In our cohort, LA volumes were underestimated by automated 3DE, which was consistent with previous studies (11). Contrary to what was found in previous publications (11, 19), automated 3DE overestimated LV volumes compared with CMR. As is mentioned above, the automated analysis software will select a best “matching” shape for the analyzed heart in its database, which is made up of various heart shapes. The surgical techniques used in our study entailed anastomoses at the mid-level of the left and right atria or at the base of the left atrial appendage (13), which makes the atria of transplanted heart bigger than the normal one. To match the big atria, the software had to choose a bigger shape than the best matching for ventricle. The biatrial group demonstrated stronger correlations for LVESV and LVEF than bicaval group after contour edit. Bicaval anastomosis technique results in varying degrees of enlargement of the two atriums, and thus the transplanted heart of the bicaval group was more twisty than the biatrial group. Therefore, it is more difficult for the software to match a suitable model and make appropriate adaption for the heart to be analyzed. We believe the difference between correlations for LVEF can be explained by the different correlations for LVESV. However, it cannot explain the similarity of the correlations for LVEDV and LA volumes between the two groups.

Our study demonstrated intraobserver and interobserver variability for LV and LA indices’ manual 3D echocardiographic measurements, which was consist with previous studies. For automated 3DE in patients without HTx, test-retest with or without contour edit, intraobserver, and interobserver variability values were similar to or slightly higher than those in previous studies (10–12, 19–21).

## Limitations

First, this was a single-center study performed in clinically well HTx recipients, and the sample size was small. Though the number of patients was enough in comparisons with the reference techniques to reach statistical significance, multi-centered studies with larger sample size are desirable to further validate the findings. In addition, the HeartModel algorithm we used was based on heart contours of patients without heart transplantation, which would inevitably hamper the accuracy of our results. We hope the database of the algorithm could add a 3DE data set of cardiac chamber images from a large cohort of HTx recipients, which could improve its feasibility and accuracy. Furthermore, lower spatial and temporal resolution in 3DE may explain in part some of the discrepancies in our results. But the mean 3DE volume rate which was 20 Hz is acceptable for the analysis of chamber morphologies.

## Conclusion

In HTx recipients, automated 3DE is feasible to a great degree. It is both reproducible and faster can be achieved than manual 3DE. Meanwhile, it is comparable with manual 3DE for measurements of left heart volumes, along with slight overestimation of LVEDV and underestimation of LAVmax, a large overestimation of LVESV and underestimation of LAVmin. But the inaccuracies for LVESV and LAVmin can be improved with manual contour edit. Although there is a good correlation between CMR and automated 3DE in LV and LA volumes from the echocardiograms of HTx recipients using the HeartModel, only LVEDV and LAVmax measured by automated 3DE with contour edit seems sufficiently accurate for clinical practice. There was slight overestimation of LVEDV and underestimation of LAVmax, but with clinically acceptable precision.

## Data Availability Statement

The original contributions presented in the study are included in the article/supplementary material, further inquiries can be directed to the corresponding authors.

## Ethics Statement

The study was approved by the Ethics Committee of Tongji Medical College, Huazhong University of Science and Technology. Furthermore, we obtained written informed consent from all participants.

## Author Contributions

YwZ, CW, YL, LZ, and MX: conception and design of the study. YwZ, CW, WS, SZ, YeZ, and YaZ: acquisition of data. YwZ and YTZ: analysis and interpretation of data. YwZ, YX, and ZZ: drafting the article. LZ: revising the article. LZ and MX: finale approval of the article. All authors listed have made a substantial, direct, and intellectual contribution to the work, and approved it for publication.

## Conflict of Interest

The authors declare that the research was conducted in the absence of any commercial or financial relationships that could be construed as a potential conflict of interest.

## Publisher’s Note

All claims expressed in this article are solely those of the authors and do not necessarily represent those of their affiliated organizations, or those of the publisher, the editors and the reviewers. Any product that may be evaluated in this article, or claim that may be made by its manufacturer, is not guaranteed or endorsed by the publisher.
